# Use of Bacopa monnieri in the Treatment of Dementia Due to Alzheimer Disease: Systematic Review of Randomized Controlled Trials

**DOI:** 10.2196/38542

**Published:** 2022-08-01

**Authors:** Aneesh Basheer, Ayush Agarwal, Biswamohan Mishra, Anu Gupta, Madakasira Vasantha Padma Srivastava, Richard Kirubakaran, Venugopalan Vishnu

**Affiliations:** 1 Department of Medicine, DM Wayanad Institute of Medical Sciences Wayanad India; 2 Department of Neurology, All India Institute of Medical Sciences New Delhi India; 3 Center for Biostatistics and Evidence Based Medicine, Christian Medical College Vellore India

**Keywords:** Bacopa monnieri, Brahmi, Ayurveda, Ayurvedic, alternative medicine, traditional medicine, complementary medicine, herb, dementia, systematic review, Alzheimer’s disease, Alzheimer disease, mild cognitive impairment, cognition, neuroprotection, memory, cognition, cognitive, treatment, therapy, clinical outcome, memory scale, neurology, neurodegenerative disease, randomized controlled trial, RCT

## Abstract

**Background:**

*Bacopa monnieri*, a herb that has been used for many centuries in India, has shown neuroprotective effects in animal and in vitro studies; human studies on patients with Alzheimer disease have been inconclusive.

**Objective:**

The primary objective of this review was to determine the clinical efficacy and safety of *B. monnieri* in persons with mild, moderate, or severe dementia, or mild cognitive impairment, due to Alzheimer disease.

**Methods:**

We searched PubMed, Embase, Cochrane Library, clinical trial registries (World Health Organization, Australia-New Zealand, United States, and South Africa), the metaRegister of Controlled Trials, and CINAHL. We intended to include all randomized and quasi-randomized controlled trials that compared *B. monnieri*, its extract or active ingredients (at any dosage), with a placebo or a cholinesterase inhibitor among adults with dementia due to Alzheimer disease and in those with mild cognitive impairment due to Alzheimer disease.

**Results:**

Our comprehensive search yielded 5 eligible studies. A total of 3 studies used *B. monnieri* in combination with herbal extracts while the remaining 2 used *B. monnieri* extracts only. Two studies compared *B. monnieri* with donepezil while the others used a placebo as the control. There was considerable variation in the *B. monnieri* dose used (ranging between 125 mg to 500 mg twice daily) and heterogeneity in treatment duration, follow-up, and outcomes. The major outcomes were Mini-Mental State Examination scores reported in 3 trials, Cognitive subscale scores of the Alzheimer’s Disease Assessment Scale in 1 study, and a battery of cognitive tests in 2 studies. Using the Cochrane risk-of-bias tool, overall, we judged all 5 studies to be at high risk of bias. While all studies reported a statistically significant difference between *B. monnieri* and the comparator in at least one outcome, we rated the overall quality of evidence for the Alzheimer’s Disease Assessment Scale-Cognitive Subscale, Postgraduate Institute Memory Scale, Mini-Mental State Examination, and Wechsler Memory Scale to be very low due to downgrading by 2 levels for high risk of bias and 1 more level for impreciseness due to small sample sizes and wide CIs.

**Conclusions:**

There was no difference between *B. monnieri* and the placebo or donepezil in the treatment of Alzheimer disease based on very low certainty evidence. No major safety issues were reported in the included trials. Future randomized controlled trials should aim to recruit more participants and report clinically meaningful outcomes.

**Trial Registration:**

PROSPERO CRD42020169421; https://www.crd.york.ac.uk/prospero/display_record.php?RecordID=169421

## Introduction

### Background

Alzheimer disease is the most common cause of dementia worldwide [1]. It is estimated that, in persons aged 65 years and above, mild cognitive impairment is present in up to 20% and dementia or major cognitive impairment is present in approximately 1% to 25% [2]. The number of people with dementia is likely to reach 44 million worldwide by 2030 [3]. The current major treatment for Alzheimer disease is drug therapy with an acetylcholinesterase inhibitor, such as donepezil [1]. These types of drugs have been found to be effective in treating dementia of all severities; however, they have no proven disease-modifying effect and, therefore, do not improve long-term outcomes [3]. In addition, their effect on patients with mild cognitive impairment due to Alzheimer disease (MCI-AD) is unproven. Furthermore, their use has been associated with adverse events such as nausea, vomiting, diarrhea, muscle spasms, insomnia, abnormal dreams, headaches, peripheral edema, weight loss, syncope, fatigue, asthenia, and tremors [4].

The search for other effective and safe treatments has led to an interest in herbs and extracts that have anecdotally been used to prevent and treat memory loss. Among many such herbs, *Bacopa monnieri* (also known as Brahmi, bacopa, or water hyssop) has attracted much attention [5-7]. This herb has been mentioned and extensively used for many centuries in Ayurveda (traditional Indian system of complementary medicine) for improving cognitive ability and preventing memory loss [8,9].

Several in vitro and animal studies have shown a neuroprotective effect of this plant and its extracts [6,10-12]. However, the use of this herb in humans has yielded conflicting results, and its role in treating patients with established Alzheimer disease is not clear. In addition, most studies [13-15] have been performed on healthy adults. Many studies (eg, [16]) from the Ayurveda stream have been anecdotal, and many others (eg, [17]) have methodologically weak designs. Systematic reviews [13,18-20] on *B. monnieri* have included studies on healthy individuals and have not included studies conducted in the last 5 years.

### How the Treatment Might Work

The major active substances in *B. monnieri* with potential effects on cognition and memory are bacoside-A, bacoside-B, alkaloids, and flavonoids [5]. The therapeutic effects of bacosides demonstrated in preclinical and in vitro studies include enhancement of neurotransmission, potentiation of synaptic activity, and repair of damaged neurons by upregulating neuronal synthesis and kinase activity [6]. Holcomb et al [10] found a reduction in amyloid levels using *B. monnieri* extract in mice models. Cognitive enhancement and neuroprotective effects have been found in Alzheimer disease animal models [21-24]. Studies have found that amnesia induced by diazepam in mice could be reversed using *B. monnieri* [25] and that L-arginine N(omega)-nitro-L-arginine–induced anterograde and retrograde amnesia could be reversed using *B. monnieri* [26]. Recently, changes in metabolites in plasma, urine, and feces in healthy individuals after consuming *B. monnieri* essence for 12 weeks were found using liquid chromatography mass spectrometry and that aminoacyl-transfer RNA, aromatic amino acids, and branched-chain amino acid biosynthetic pathways were mainly related to the metabolites identified in all 3 types of samples [27]. Aromatic amino acids, particularly phenylalanine, are metabolites found to decrease in level in the plasma of healthy community-dwelling participants aged 70 years and older who later progressed to amnestic mild cognitive impairment or Alzheimer disease compared to normal controls [28].

Considering the potential of *B. monnieri* as a neuroprotective agent and the gap in the existing literature connecting *B. monnieri* and dementia, we aimed to perform a systematic review to determine whether it has beneficial effects on cognitive impairment due to Alzheimer disease and identify gaps in the literature.

### Review Question and Objectives

We aimed to address the following question: What are the effects (benefits and harms) of *B. monnieri* on individuals with dementia due to Alzheimer disease?

Our objectives were to (1) determine the clinical efficacy and safety of *B. monnieri* in persons with mild, moderate, or severe dementia due to Alzheimer disease or with MCI-AD; and (2) compare the efficacy and safety of different doses of *B. monnieri*.

## Methods

### Search Strategy

A comprehensive search strategy (Multimedia Appendix 1) was employed to identify all relevant studies. We did not place any restrictions on language or publication status (published and in press) during the search. PubMed, Embase, Cochrane Library, clinical trial registries (World Health Organization, Australia-New Zealand, United States, and South Africa), the metaRegister of Controlled Trials, and CINAHL were searched from inception to January 2021. We also searched for studies in the reference lists of all studies included in the pool of retrieved papers. Where possible, we contacted authors to obtain the full text of the papers. The study has been reported according to the PRISMA (Preferred Reporting Items for Systematic Reviews and Meta-Analyses) guidelines, and the protocol is available on the International Prospective Register of Systematic Reviews (CRD42020169421).

### Study Selection

We included randomized and quasi-randomized controlled trials that compared *B. monnieri*, its extract or active ingredients (at any dosage), with a placebo or a cholinesterase inhibitor among adults with dementia due to Alzheimer disease and MCI-AD. Cohort studies, case-control studies, case reports, systematic reviews, policy papers, letters to the editor, correspondence, and nonhuman studies were excluded. We also excluded trials that were confounded by treatment or a control group receiving another active treatment that has not been factored into the randomization. Furthermore, studies must report one or more of the following outcomes to be eligible for inclusion: cognitive function (determined by the change from baseline in the Alzheimer’s Disease Assessment Scale-Cognitive Subscale (ADAS-Cog), Mini-Mental State Examination, Postgraduate Institute Memory Scale, or any culturally adapted or validated tools to assess cognition), activities of daily living using scores such as the Alzheimer Disease Cooperative Study, clinician-rated global impression tests, behavioral symptoms, and safety as measured by incidence of adverse effects, dependency, or death.

### Data Extraction and Quality Assessment

Following the search, 2 authors (VVY and AB) screened titles, abstracts, and full-text papers independently and extracted data from each paper using standardized data extraction forms. Discrepancies were resolved by consensus between the 2 authors or by a third author (AA). Data on study characteristics, participant characteristics (age, gender, healthy adults, patients with Alzheimer disease), study setting, use of *B. monnieri* (formulation, dose), placebo, any active comparator, and outcomes of interest were extracted. Two authors (AB and BM) used the Cochrane risk-of-bias tool for randomized controlled trials [29] to determine the risk of bias for all eligible papers, and a third author (AA) independently assessed the papers that were found to be eligible to be included in this systematic review in terms of the level of concern (ie, low, some, or high). We did not group studies comparing *B. monnieri* with the placebo and those comparing *B. monnieri* with cholinesterase inhibitors since no meaningful comparisons were possible due to the heterogeneity of outcomes. Since none of the outcomes of interest was reported for all studies, we did not pool them during analysis.

### Data Analysis

We used ReviewManager (version 5.4; The Cochrane Collaboration) for data analysis. Wherever possible, an intention-to-treat analysis was planned. Due to explicit clinical and methodological heterogeneity between available studies, we performed no statistical tests for heterogeneity. The data available from eligible studies precluded a meta-analysis, and we performed an interpretative synthesis of data from individual studies. The GRADE (Grading of Recommendations Assessment, Development and Evaluation) framework was used to summarize the certainty of evidence [30,31]. We did not attempt to ascertain publication bias since there were only a few eligible studies.

## Results

### Overview

**Figure 1:**
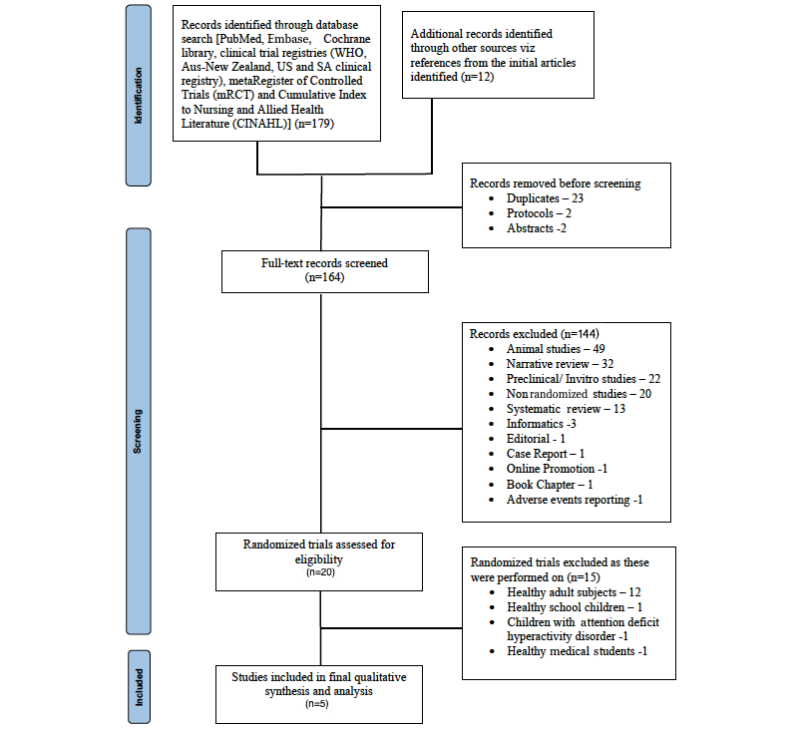
PRISMA (Preferred Reporting Items for Systematic Reviews and Meta-Analyses) flowchart showing the identification, screening, and inclusion of trials for this systematic review. WHO: World Health Organization, US: United States, SA: South Africa.

Overall, 164 studies were identified by the search. The abstracts of all studies were screened, and 5 [32-36] were found to be eligible (Figure 1 and Multimedia Appendices 2 and 3).

The primary objective of the study conducted by Prabhakar et al [32] was to determine if *B. monnieri* improved the memory of patients with Alzheimer disease and MCI-AD compared with donepezil [32]. The diagnosis of Alzheimer disease and MCI-AD was aided by magnetic resonance imaging of the brain, fluorodeoxyglucose-positron emission tomography of the brain, and cerebrospinal fluid biomarkers (beta amyloid and total tau). *B. monnieri* was administered once daily at a dose of 300 mg, while 10 mg of donepezil was given once daily for 12 months. Although a sample size of 48 patients (24 in each arm) was planned, only 34 patients (17 in each arm) could be recruited after 45 months, due to which the study was terminated. The primary outcome was the difference in change of the ADAS-Cog score and Postgraduate Institute Memory Scale score from baseline after 12 months of treatment between the 2 treatment groups. However, patients were followed up for changes in scores at 3, 6, and 9 months of treatment. Change from baseline of neuropsychological tests such as the verbal fluency-controlled oral word test and animal names test, quality of life-Alzheimer disease, activities of daily living inventory, adherence to treatment, and adverse events were secondary outcomes. The authors reported attempts to follow up on all patients with whom contact was lost during the study period, and an intention-to-treat analysis was used. Missing data were handled by multiple imputations due to loss of follow-up. A total of 4 and 9 patients were lost to follow-up in the donepezil and *B. monnieri* arms, respectively, at the end of 12 months. There were differences in baseline characteristics (more patients with Alzheimer disease in the donepezil arm and more patients with MCI-AD in the *B. monnieri* arm), which were adjusted for during analyses.

Cicero et al [33] compared the effect of a combination of agents (*B. monnieri*, L-theanine, *Crocus sativus*, copper, folate, vitamin B complex, and vitamin D) over 2 months and those of a placebo in improving cognitive functions in older adult patients. They included 30 participants with Mini-Mental State Examination scores between 20 and 27 or self-perceived cognitive impairment (whether the impairment was dementia or mild cognitive impairment was not reported). The primary outcome was change in Mini-Mental State Examination score from baseline at 2 months. The Perceived Stress Questionnaire and Self-Rating Depression Scale scores were other outcomes.

Sadhu et al [34] investigated the efficacy of a polyherbal test formulation composed of extracts of *B. monnieri*, *Hippophae rhamnoides*, and *Dioscorea bulbifera* (total dose of 500 mg) on cognitive functions [34]. The test formulation was compared with a placebo in healthy adults without dementia and the test formulation was compared with donepezil (10 mg twice a day) in older adult patients (aged 60-75 years) with Alzheimer disease (n=123; deterioration of memory in at least 3 of the following: poor orientation, poor judgment and problem-solving, difficulty in community affairs, inability to function independently at home, or difficulties in personal care) for 12 months. Subsequently, the participants underwent a clinical screening using the Dementia Rating Scale-2 before being randomized to test formulation or donepezil. The primary outcome was cognitive function assessed by a composite of mental status (Mini-Mental State Examination), verbal memory, complex psychomotor tests, and attention or executive functions at 12 months.

Raghav et al [35] studied the efficacy of *B. monnieri* extracts in patients with age-associated memory impairment and no evidence of dementia or psychiatric illness [37]. Participants were adults older than 55 years of age with memory loss in daily activities and a logical subset score <6 on the Wechsler Memory Scale. Patients with a Mini-Mental State Examination score >24 were excluded. Eligible patients (N=40; *B. monnieri* group: n=20; placebo group: n=20) were randomized to receive 125 mg of *B. monnieri* extract or a placebo twice a day for 12 weeks followed by the placebo for both groups for another 4 weeks. The outcomes were the Mini-Mental State Examination and the Wechsler Memory Scale (subsets Logical Memory, Visual Reproduction, and Paired Associated Learning).

Barbhaiya et al [36] studied the effects of Bacomind(an enriched phytochemical combination containing 450 mg of standardized *B. monnieri* extract) on 65 individuals aged 50 to 75 years with self-reported memory impairment (Mini-Mental State Examination score >24) for at least 1 year. The study duration was 24 weeks (drug administered for 12 weeks, then no drugs for 12 weeks). Neuropsychological assessment was performed at baseline, week 12, and week 24. Outcomes were analyzed for attention, memory, and speed of information processing. Three patients were lost to follow-up for the second visit, and 15 patients were removed as outliers. The final analysis was completed for 44 patients (Bacomind: n=23; placebo: n=21). Further, 3 patients were lost to follow-up during the study.

### Risk of Bias

#### Random Sequence Generation and Allocation

We judged all 5 studies to be at low risk of bias in terms of random sequence generation (Multimedia Appendix 4).

For allocation concealment, a judgment of low risk of bias was made for all studies except Raghav et al [35], which was considered as having some concerns. Barbhaiya et al [36] provided the drug and the placebo in coded bottles. Prabhakar et al [32], Cicero et al [33], and Sadhu et al [34] described the concealment of allocation in sufficient detail, while in the study by Raghav et al [35], the actual method of allocation concealment was unclear.

#### Deviations From the Intended Interventions

All 5 studies reported that they were double-blinded. Prabhakar et al [32] and Barbhaiya et al [36] described the use of identical-looking capsules as a placebo, while Cicero et al [33] described how the blinding of interventions was ensured throughout the study. Raghav et al [35] did not describe how blinding was performed or ensured till the end of the study. Sadhu et al [34] provided no details on the blinding process and how it was maintained throughout the study. Prabhakar et al [32] and Barbhaiya et al [36] used coded medicines in strips or bottles, respectively. None of the trials reported any deviations from intended interventions due to trial context.

Prabhakar et al [32] had envisaged an original sample size of 48; however, they could only recruit 34 patients (17 in each group). Finally, at the end of the study, only 13 remained in the donepezil group and 8 remained in the *B. monnieri* group (24% and 53% loss to follow-up, respectively). Although they described using intention-to-treat analysis, the loss to follow-up was considered sufficiently significant to be judged as a high risk of bias.

There was no loss to follow-up in the study by Cicero et al [33], and we considered it to be at low risk of bias. Sadhu et al [34] reported that out of 123 patients who took part in the study, 104 completed the study (15% lost to follow-up). However, there was no mention of how missing data were handled or if an intention-to-treat analysis was used. Hence, this study was also considered to have some concerns regarding the risk of bias. Raghav et al [35] reported that 5 out of 40 participants dropped out of the study. They used a per-protocol analysis and did not report how missing data were handled or the use of intention-to-treat analysis. We judged it to be at high risk of bias. Barbhaiya et al [36] reported a 32% loss to follow-up (21 out of 65 patients), out of which 15 patients were removed as outliers. Neither a definition for outliers nor an explanation for the patients’ removal was given. Barbhaiya et al [36] used a per-protocol analysis and did not mention how missing data were handled. Hence, we judged this study to be at high risk of bias in this domain.

#### Missing Outcome Data

In this domain, we judged Cicero et al [33] to have a low risk of bias while all other studies were at high risk of bias considering that outcome data were not available for nearly all participants in these studies and that this missing data could have potentially biased the results.

#### Measurement of Outcome

Prabhakar et al [32] and Sadhu et al [34] were considered to have a low risk of bias for this domain. Cicero et al [33], Raghav et al [35], and Barbhaiya et al [36] were judged as having a high risk of bias considering that no information was available on the blinding of outcome assessors that could have potentially biased results.

#### Selection of the Reported Results

All 5 studies reported outcomes stated in their methods or protocol. However, only Prabhakar et al [32] reported the primary outcome of interest (ADAS-Cog). Overall, we judged all studies to be at low risk of bias for this domain.

#### Other Potential Sources of Bias

Prabhakar et al [32] had amended their protocol regarding the timing of primary outcome assessment. In their initial protocol, the primary outcomes were supposed to be assessed at 3, 6, 9, and 12 months. In their amendment, they had fixed the timeline at 12 months alone for the primary outcome. The primary outcome assessment at 12 months was delayed for many patients.

Sadhu et al [34] included healthy participants and patients with Alzheimer disease in the study; however, the healthy participants had low baseline Mini-Mental State Examination scores (group A, healthy older adult participants receiving a placebo: mean 17.48, SD 3.72; group B, healthy older adult participants receiving the test formulation: mean 16.93, SD 3.71) and cannot be considered healthy. Scores for the patients with Alzheimer disease were very low (group C, patients with senile dementia of the Alzheimer’s type [SDAT] given donepezil 10 mg twice daily: mean 7.019, SD 1.316; group D, SDAT patients given the test formulation twice daily: mean 6.014, SD 1.212). The test formulation contained extracts of *B. monnieri* (whole plant), *Hippophae rhamnoides* (leaves and fruits), and *Dioscorea bulbifera* (bulbils). At baseline, among the healthy participants, 9 had psychotic features and 25 had depression. In the Alzheimer disease group, 68 had depression and 67 had psychotic features. Neuroimaging was not mentioned. In addition, there was a discrepancy in the reported outcomes: The text states that there was no significant improvement in immediate word recall, attention span, Functional Activity Questionnaire score, or depression scores at 12 months; however, in the tables, all these subsets of cognitive evaluation mentioned having statistically significant improvement. In the study by Raghav et al [35], the study duration was 3 months, and in that short period, there was a significant improvement with regards to the subsets Logical Memory, Mental Control, Digit Forward, and Paired Associate Learning, even in the placebo group.

Prabhakar et al [32] had registered their trial protocol in Clinical Trials Registry - India retrospectively. None of the other trials were registered. The diagnostic certainty of Alzheimer disease was high only in Prabhakar et al [32].

### Overall Bias

All 5 studies were deemed to have a high risk of bias.

#### Effects of Intervention

None of the studies described the effects of *B. monnieri* in patients with different classes of disease severity (mild, moderate, severe Alzheimer disease and MCI-AD). Effects of different dosages of *B. monnieri* were not tested in any eligible studies. Hence, the primary and secondary objectives of the review remain unanswered.

#### Cognitive Functions

Only Prabhakar et al [32] reported effects on cognition using ADAS-Cog and Postgraduate Institute Memory Scale. Prabhakar et al [32], Sadhu et al [34], and Cicero et al [33] reported changes in Mini-Mental Status Examination; although Raghav et al [35] mentioned that Mini-Mental State Examination was performed at baseline in all participants, we could not extract data about the change in scores. Barbhaiya et al [36] used Mini-Mental State Examination only for screening.

Prabhakar et al [32] performed an intention-to-treat analysis; after adjustment for confounders, no difference in the rate of change in the ADAS-Cog score was noted between the *B. monnieri* arm and donepezil arm at any of the prespecified time points (3, 6, 9, and 12 months) from baseline. At 12 months, the mean ADAS-Cog score was 2.27 (SD 5.65) in the donepezil arm and 0.51 (SD 5.65) in the *B. monnieri* arm (mean difference –1.76; *P*=.39). There was a significant difference in the change in overall Postgraduate Institute Memory Scale score between the 2 arms at 12 months (donepezil: mean 0.46; SD 10.96; *B. monnieri*: mean 7.94, SD 10.96); mean difference –8.40; *P*=.04]. The donepezil arm had reduced progression of symptoms (measured by ADAS‑Cog scores) in individuals with MCI‑AD or mild‑to‑moderate AD, compared to those in the *B. monnieri* arm. However, analysis of individual components of the Postgraduate Institute Memory Scale revealed no differences.

Changes in Mini-Mental State Examination scores from baseline were reported by Sadhu et al [34], Cicero et al [33], and Prabhakar et al [32]. At 3 months, a significant difference in the mean change from baseline was noted between the donepezil arm (mean 0.72, SD 3.13) and the *B. monnieri* arm (mean –2.02, SD 3.13) by Prabhakar et al [32] (mean difference 2.74; *P*=.02); however, there were no differences between the arms at any further time points (6, 9, and 12 months). Sadhu et al [34] also found no difference between the donepezil arm (mean 7.882, SD 1.956) and the *B. monnieri* formulation arm (mean 7.914, SD 2.106) at 12 months in terms of change in Mini-Mental State Examination scores from baseline (*P*=.9375).

Cicero et al [33] found significant improvements in the Mini-Mental State Examination score and the Perceived Stress Questionnaire index in the *B. monnieri* formulation arm compared to the placebo. Both were reported as mean scores before and after treatment.

Raghav et al [35] used the Wechsler Memory Scale to report outcomes. They reported scores of individual subsets of the scale at baseline, 4, 8, 12, and 16 weeks as means and standard deviations. The total memory score of the *B. monnieri* arm showed a significant difference from the placebo arm in terms of change from baseline at 4, 8, and 12 weeks but not at 16 weeks. Raghav et al [35] also reported that 55% of participants in the *B. monnieri* arm showed more than 20% improvement in memory parameters compared with 44.4% of participants in the placebo arm (*P*<.01).

Barbhaiya et al [36] used various tests for attention (digit span, digit cancellation, serial subtraction), memory (Rey Auditory Verbal Learning Test, Wechsler Memory Scale-1, paired associates, and visual retention), speed of information processing (digital symbols). There was a significant improvement in the digit span backward task (*P*=.008) and digit cancellation time test (*P*<.001) between baseline and week 12. A significant improvement in list learning delayed recall (*P*=.014), paired associates dissimilar delayed recall (*P*=.047), and visual retention test (*P*=.035) were also reported.

### Functional Outcomes

Different tools were used in the studies to determine functional outcomes. Prabhakar et al [32] found no significant difference in the change in activities of daily living scores such as Alzheimer Disease Cooperative Study activities of daily living at any time during follow-up between the donepezil and *B. monnieri* arms. Similarly, no changes were noted in quality of life measured using Quality of Life Patient and Informant questionnaires. Sadhu et al [34] used the Functional Activity Questionnaire and reported a significant difference in change at 12 months between the donepezil arm (mean 9.801, SD 1.458) and the *B. monnieri* formulation arm (mean 11.873, SD 2.751; *P*<.001). Raghav et al [35] did not report any functional or quality of life–related outcomes. Cicero et al [33] reported significant improvement in Self-Rating Depression Scale scores in the *B. monnieri* formulation arm. Barbhaiya et al [36] did not report any functional outcomes.

### Safety

Prabhakar et al [32] reported no significant differences in the number of patients who experienced one or more adverse events. No major adverse events were reported either. There were 3 deaths (2 in donepezil arm and one in *B. monnieri* arm) reported due to myocardial infarction. Raghav et al [35] reported diarrhea in 1 patient and headache in 2 patients in the placebo arm. One participant of the *B. monnieri* arm was reported to have experienced rashes. Sadhu et al [34] reported nausea, constipation, and drowsiness; however, no data on the number of participants with these events were reported. In the study conducted by Cicero et al [33], 1 participant was reported to have an aftertaste following ingestion of the *B. monnieri* formulation. Barbhaiya et al [36] did not report any adverse events.

### Quality of the Evidence

We used the GRADE (Grading of Recommendations Assessment, Development and Evaluation) approach to assess the certainty of the evidence in the included studies, using the criteria outlined in the Cochrane Handbook [37]. The certainty of the evidence for the reported outcomes was very low. We assessed the quality of evidence for 4 major clinically relevant outcomes (ADAS-Cog, Postgraduate Institute Memory Scale, Mini-Mental State Examination, and Wechsler Memory Scale). We judged that all 5 included studies had a high risk of bias, downgrading the evidence by 2 levels. We downgraded one level for impreciseness due to the small sample size and wide CI. Hence, the overall certainty of evidence was very low.

## Discussion

### Principal Results

There was no high-quality evidence for the benefits of *B. monnieri* compared with a placebo or donepezil for cognitive function, functional outcomes, or adverse events. All 5 studies were heterogeneous with respect to doses of *B. monnieri*, *B. monnieri* as part of a polyherbal combination, use of a placebo or donepezil as the control group, duration of treatment (2 months to 12 months), cognitive tests to assess primary outcomes, and the diagnostic criteria for Alzheimer disease and mild cognitive impairment.

### Lack of Neuroimaging for the Diagnosis

Prabhakar et al [32] was able to demonstrate a statistically better outcome in the donepezil group compared to the *B. monnieri* group, even though there was no difference at 3, 6, and 9 months. Any statistical significance in this study was limited by a small sample size (34 patients) due to poor recruitment (the trial was stopped prematurely because of this issue).

Raghav et al [35], Sadhu et al [34], and Cicero et al [33] were able to demonstrate improvements in one or more facets of cognition using *B. monnieri* compared to the placebo or donepezil. All 3 studies had small sample sizes. Furthermore, duration of treatment and follow-up were also short. Since Sadhu et al [34] and Cicero et al [33] used a polyherbal preparation and combined nutraceuticals (with *B. monnieri* as one component), improvements noted cannot be attributed to *B. monnieri* alone. These 2 studies [33,34] did not use brain imaging; hence, the accuracy of Alzheimer disease diagnosis cannot be ascertained.

Although Barbhaiya et al [36] reported significant improvements in several tests of cognition, removal of 15 participants after randomization as outliers without giving any reasonable explanation and the absence of 33% of participants from the final analysis are major issues.

### Comparison With Other Studies or Reviews

A previous meta-analysis [13] that evaluated the efficacy of *B. monnieri* for cognitive performance included studies with both healthy participants and individuals with memory impairment (518 participants from 9 studies); however, there were only 2 trials with cognitively impaired patients [35,36]. Similar to our observation, Barbhaiya et al [36] described an overall dropout of around 33%. However, there is no mention of the exclusion of 15 participants as outliers. A meta-analysis performed on data from 437 participants showed a shortened duration taken to complete the Trail B test (–17.9 ms, 95% CI –24.6 to –11.2; *P*<.001) and decreased choice reaction time (10.6 ms, 95% CI –12.1 to –9.2; *P*<.001). However, the Trail B test results were based on a single study with 46 healthy volunteers while the decreased choice reaction time was based on a subgroup analysis of 2 studies on healthy volunteers (46 and 62 participants) using 300 mg of *B. monnieri*. Hence, it cannot be truly considered as pooled estimates of efficacy in those with dementia. We did not perform a meta-analysis since the heterogeneity of data from the studies on people with dementia precluded meaningful pooling.

Another systematic review [18] on the effectiveness of *B. monnieri* as a nootropic, neuroprotective, or antidepressant supplement included studies involving healthy volunteers and those with dementia and depression. Three studies evaluated *B. monnieri* in Alzheimer disease or mild cognitive impairment [33,34,38], of which Goswami et al [38] was a nonrandomized study. A meta-analysis was not performed in this review.

In a systematic review by Cicero et al [19], the meta-analysis by Kongkeaw et al [13] was cited, and no new studies were included apart from those included by Kongkeaw and colleagues. Brioschi Guevara et al [20] had included 2 studies (Raghav et al [35] and Barbhaiya et al [36]) in their systematic review and described these studies as follows: “two old small studies on *B. monnieri* in individuals with memory complaints suggest a potential effect on some aspect of memory function or on attention tests that still need to be confirmed.” No data or study characteristics were mentioned in the review.

Though previous systematic reviews [13,19] had included most of the studies that this review also found eligible, they had not specifically addressed the question of the efficacy of *B. monnieri* in persons with Alzheimer disease. Inclusion of healthy volunteer studies and nonrandomized studies in these reviews makes it difficult to draw conclusions about similarities, and although significant improvements in specific tests had been noted in pooled analyses, they were mostly based on data from subgroups or single studies.

### Strengths

We have thoroughly searched for and analyzed all the available evidence critically. Only 5 eligible trials were identified due to the use of stringent inclusion criteria. Due to severe heterogeneity in the included studies in terms of criteria for diagnosis, cognitive tests used, *B. monnieri* formulations (including polyherbal), duration of treatment, and lack of confidence in the diagnosis of dementia in the included patients, we did not conduct a meta-analysis.

### Limitations

First, the use of very stringent inclusion criteria led to few eligible trials. However, this also means that the review question has been addressed specifically without any dilution of intent. Second, 1 author (VVY) was involved in a trial included in this review (ie, Prabhakar et al [32]). This potential source of bias was addressed because author VVY did not participate in the risk-of-bias assessment of trials (performed independently by authors AB and BM). Third, as with any systematic review, it is possible that some studies might have been missed. We ensured the inclusion of all potential studies by searching multiple databases. Moreover, independent screening of search output by 2 authors ensured that bias was minimized in assessing eligibility. We followed the guidance provided in the Cochrane Handbook to minimize potential biases in the review process [34]. Fourth, because only 5 trials were included in this review, we could not use funnel plots to assess the risk of publication bias.

### Future Directions

As discussed above, this review found that there was no high-quality evidence for the benefits of *B. monnieri* compared with a placebo or donepezil for cognitive function, functional outcomes, or adverse events. All 5 studies had heterogeneity with respect to the *B. monnieri* dosage used in the trials, *B. monnieri* used as part of a polyherbal combination, use of a placebo or donepezil as the control group, duration of treatment (2 months to 12 months), cognitive tests used to assess primary outcomes, and diagnostic criteria used for Alzheimer disease and mild cognitive impairment. These hindered the generation of high-quality evidence for the use of *B. monnieri* in Alzheimer disease and MCI-AD*.* Based on these results, we opine the following design changes for future trials of *B. monnieri* in patients with Alzheimer disease.

#### Is B. monnieri Studied as a Disease-Modifying Drug or Only as a Symptomatic Treatment?

If the drug is tested as a disease-modifying treatment, then a placebo-controlled trial should be performed with stratification for the use of symptomatic medications (such as cholinesterase inhibitors) undertaken at randomization. If the drug has only a symptomatic effect, then it can either be tested against a placebo or with a cholinesterase inhibitor that is the standard of care for symptomatic management in many countries.

#### Drug Dosage

There is wide variation in the dosage of *B. monnieri* used in clinical trials. In the review by Kongkeaw et al [13], the most commonly used dosage was 300 mg twice daily. Our review also included studies where different doses of *B. monnieri* were used. The most commonly used dosage was again 300 mg twice daily. The analysis revealed no difference between *B. monnieri* and a placebo or donepezil in the treatment of Alzheimer disease or mild cognitive impairment. Both 300 mg and 450 mg of *B. monnieri* have been reported to be safe in a phase 1 study [39]. Hence, future trials of *B. monnieri* may consider using the higher dose (ie, 450 mg, twice daily) for efficacy. Since many studies use polyherbal preparations (eg, [40]), it is difficult to ascertain the role of each constituent.

#### Diagnosis of Dementia

The inclusion criteria for participants should be clearly defined, and internationally accepted criteria for dementia should be used. The International Working Group criteria and the National Institute on Aging –Alzheimer's Association criteria describe 3 stages in the Alzheimer disease continuum (preclinical Alzheimer disease, prodromal Alzheimer disease or MCI-AD, and Alzheimer disease dementia) [41].

#### Duration of Treatment

The trial duration depends on the intervention strategy (primary prevention vs secondary prevention), target population (preclinical Alzheimer disease and prodromal or MCI-AD vs established Alzheimer disease dementia), efficacy endpoints (cognition, functional, or global endpoints; behavioral; and psychiatric symptoms of dementia), and mechanism of intervention (symptomatic treatment vs disease-modifying treatment). Prevention trials require a minimum duration of 3 years. In patients with mild to moderate and prodromal Alzheimer disease or MCI-AD, a minimum duration of 18 months has been assumed to be sufficient [42]; longer durations might be necessary depending on the timing of the intervention and trial design (eg, delayed start or time to event approach). For symptomatic treatment of behavioral and psychiatric symptoms of dementia in established dementia, a duration of 8 to 12 weeks is recommended [42].

#### Effect Size Estimates and Sample Size

Since in most of the included trials, the diagnosis of Alzheimer disease itself was not conclusive and different outcome measures were used, it is difficult to estimate the required sample size for a future study. We would suggest that internationally accepted and harmonized clinical outcome measures such as the ADAS-Cog should be used in a future trial, with 2 caveats. First, the cross-cultural validity of the tool being used needs to be considered, and second, the possibility of a ceiling effect in prodromal (pre–mild cognitive impairment and mild cognitive impairment stages) Alzheimer disease should also be taken into account [43]. For the latter issue, studies are now using tools that are sensitive to detect changes in cognitively less impaired individuals and can capture the earliest clinically meaningful changes over a respectable time duration of the trial [44]. Emphasis is on the creation and validation of cognitive composite scores (eg, a composite score including delayed word list recall, logical memory, category fluency, tests of processing speed, tests of performance IQ) as primary efficacy measures in Alzheimer disease prevention trials to detect subtle cognitive changes between treatment and placebo groups [45]. There are several studies [46,47] on power calculations of clinical trials on Alzheimer disease.

Here, we consider the Alzheimer Disease Neuroimaging Database and its power calculations for sample size estimation [46,48]. The effect size can be estimated as a percentage of the anticipated mean rate of decline under the placebo or standard-of-care scenario. The sample size required to detect a 25% reduction in the annual rate of change for the ADAS-Cog in mild cognitive impairment (80% power and 2-sided α=.05), with an annual rate of change of 2.5 points is 1183 for a 1-year trial; however, if the estimate is for a 25% reduction of a 1.0-point rate of change per year, the sample size would be 2175. A similar calculation for patients with Alzheimer disease will yield a sample size from 312 to 624 per arm for a 1-year trial, assuming a 25% reduction of 3.8 to 4.3 points per year. Only Prabhakar et al [32] used the ADAS-Cog as their primary outcome, but data are not available separately for patients with mild cognitive impairment and Alzheimer disease; the annual rate of change of the score for the donepezil arm was 2.2 points. Hence, it is reasonable to assume annual rates of decline of 2.5 and 3.8 points in the placebo arm for mild cognitive impairment and Alzheimer disease, respectively; the required sample size for a 25% reduction in score in the *B. monnieri* treatment arms would be 1183 and 500 for mild cognitive impairment and Alzheimer disease, respectively, for a 1-year trial. The sample size can be reduced if a surrogate outcome like hippocampal atrophy is used. Using similar calculations as above, the sample size for mild cognitive impairment will be around 200 to 300 per arm for a 1-year trial if the assumed annual rate of hippocampal atrophy is 2% to 3%. In Alzheimer disease, the sample size will be much less if we consider a 50% slowing of overall hippocampal atrophy; then the sample size can be reduced to less than 100 participants.

#### Outcome Measures

Primary outcomes for Alzheimer disease trials should include cognitive and functional endpoints or a single cognition-function composite endpoint, and the tools selected to capture these outcomes should have cultural validity and international harmony. Prodromal Alzheimer disease assessment requires newer, sensitive measures, such as tests of metacognition, social cognition, and prospective memory [43], rather than traditional neuropsychological tests. The detection of functional impairment in the early stages will also require instruments that are sensitive to subtle functional changes such as tests for financial capacity, performance-based skill assessments, and computerized assessments based on virtual reality and video technology [43]. Other options are time to onset of dementia or the proportion of patients who develop Alzheimer disease dementia; however, Alzheimer disease prevention trials that use time to event as an outcome require extended observation periods to accurately assess disease progression [49].

### Conclusions

The evidence obtained from the present systematic review is of very low certainty. The evidence from 5 trials suggests that there is no difference between *B. monnieri* and a placebo or donepezil in the treatment of Alzheimer disease or mild cognitive impairment. No major safety issues were reported in the trials included in this review.

## Appendix

Multimedia Appendix 1 Search strategy.

Multimedia Appendix 2 Detailed baseline characteristics of the included studies.

Multimedia Appendix 3 Quality of evidence, outcomes, and adverse events.

Multimedia Appendix 4 Risk-of-bias assessment.

## Abbreviations

ADAS-Cog: Alzheimer’s Disease Assessment Scale-Cognitive Subscale
GRADE: Grading of Recommendations Assessment, Development, and Evaluation
MCI-AD: mild cognitive impairment due to Alzheimer disease
PRISMA: Preferred Reporting Items for Systematic Reviews and Meta-Analyses
SDAT: senile dementia of the Alzheimer’s type

## References

[1] Rabinovici GD (2019). Late-onset Alzheimer Disease. CONTINUUM: Lifelong Learning in Neurology.

[2] Petersen RC (2016). Mild Cognitive Impairment. CONTINUUM: Lifelong Learning in Neurology.

[3] Livingston G, Sommerlad A, Orgeta V, Costafreda SG, Huntley J, Ames D, Ballard C, Banerjee S, Burns A, Cohen-Mansfield J, Cooper C, Fox N, Gitlin LN, Howard R, Kales HC, Larson EB, Ritchie K, Rockwood K, Sampson EL, Samus Q, Schneider LS, Selbæk G, Teri L, Mukadam N (2017). Dementia prevention, intervention, and care. The Lancet.

[4] Colovic MB, Krstic DZ, Lazarevic-Pasti TD, Bondzic AM, Vasic VM (2013). Acetylcholinesterase Inhibitors: Pharmacology and Toxicology. CN.

[5] Singh HK, Dhawan BN (1997). Neuropsychopharmacological effects of the Ayurvedic nootropic Bacopa monniera Linn (Brahmi). Indian J Pharmacol.

[6] Mathur D, Goyal K, Koul V, Anand A (2016). The Molecular Links of Re-Emerging Therapy: A Review of Evidence of Brahmi (Bacopa monniera). Front Pharmacol.

[7] Roodenrys S, Booth D, Bulzomi S, Phipps A, Bulzomi S, Micallef C, Smoker J (2002). Chronic effects of Brahmi (Bacopa monnieri) on human memory. Neuropsychopharmacology.

[8] Rao Rammohan V, Descamps Olivier, John Varghese, Bredesen Dale E (2012). Ayurvedic medicinal plants for Alzheimer's disease: a review. Alzheimers Res Ther.

[9] Nemetchek Michelle D, Stierle Andrea A, Stierle Donald B, Lurie Diana I (2017). The Ayurvedic plant Bacopa monnieri inhibits inflammatory pathways in the brain. J Ethnopharmacol.

[10] Holcomb LA, Dhanasekaran M, Hitt AR, Young KA, Riggs M, Manyam BV (2006). Bacopa monniera extract reduces amyloid levels in PSAPP mice. J Alzheimers Dis.

[11] Khan M Badruzzaman, Ahmad Muzamil, Ahmad Saif, Ishrat Tauheed, Vaibhav Kumar, Khuwaja Gulrana, Islam Fakhrul (2015). Bacopa monniera ameliorates cognitive impairment and neurodegeneration induced by intracerebroventricular-streptozotocin in rat: behavioral, biochemical, immunohistochemical and histopathological evidences. Metab Brain Dis.

[12] Dwivedi Subhash, Nagarajan Rajasekar, Hanif Kashif, Siddiqui Hefazat Husain, Nath Chandishwar, Shukla Rakesh (2013). Standardized Extract of Bacopa monniera Attenuates Okadaic Acid Induced Memory Dysfunction in Rats: Effect on Nrf2 Pathway. Evid Based Complement Alternat Med.

[13] Kongkeaw C, Dilokthornsakul P, Thanarangsarit P, Limpeanchob N, Norman Scholfield C (2014). Meta-analysis of randomized controlled trials on cognitive effects of Bacopa monnieri extract. J Ethnopharmacol.

[14] Peth-Nui Tatimah, Wattanathorn Jintanaporn, Muchimapura Supaporn, Tong-Un Terdthai, Piyavhatkul Nawanant, Rangseekajee Poonsri, Ingkaninan Kornkanok, Vittaya-Areekul Sakchai (2012). Effects of 12-Week Bacopa monnieri Consumption on Attention, Cognitive Processing, Working Memory, and Functions of Both Cholinergic and Monoaminergic Systems in Healthy Elderly Volunteers. Evid Based Complement Alternat Med.

[15] Calabrese Carlo, Gregory William L, Leo Michael, Kraemer Dale, Bone Kerry, Oken Barry (2008). Effects of a standardized Bacopa monnieri extract on cognitive performance, anxiety, and depression in the elderly: a randomized, double-blind, placebo-controlled trial. J Altern Complement Med.

[16] Pandey M M, Rastogi Subha, Rawat A K S (2013). Indian traditional ayurvedic system of medicine and nutritional supplementation. Evid Based Complement Alternat Med.

[17] Sridharan Kannan, Sivaramakrishnan Gowri (2016). Clinical trials in Ayurveda: Analysis of clinical trial registry of India. J Ayurveda Integr Med.

[18] Brimson JM, Brimson S, Prasanth MI, Thitilertdecha P, Malar DS, Tencomnao T (2021). The effectiveness of Bacopa monnieri (Linn.) Wettst. as a nootropic, neuroprotective, or antidepressant supplement: analysis of the available clinical data. Sci Rep.

[19] Cicero AF, Fogacci F, Banach M (2018). Botanicals and phytochemicals active on cognitive decline: The clinical evidence. Pharmacol Res.

[20] Brioschi Guevara Andrea, Bieler Melanie, Altomare Daniele, Berthier Marcelo, Csajka Chantal, Dautricourt Sophie, Démonet Jean-François, Dodich Alessandra, Frisoni Giovanni B, Miniussi Carlo, Molinuevo José Luis, Ribaldi Federica, Scheltens Philip, Chételat Gael (2021). Protocols for cognitive enhancement. A user manual for Brain Health Services-part 5 of 6. Alzheimers Res Ther.

[21] Brimson JM, Prasanth MI, Plaingam W, Tencomnao T (2020). Bacopa monniera extract protects against glutamate toxicity and increases the longevity of. J Tradit Complement Med.

[22] Uabundit N, Wattanathorn J, Mucimapura S, Ingkaninan K (2010). Cognitive enhancement and neuroprotective effects of Bacopa monnieri in Alzheimer's disease model. J Ethnopharmacol.

[23] Saini N, Singh D, Sandhir R (2012). Neuroprotective effects of Bacopa monnieri in experimental model of dementia. Neurochem Res.

[24] Saraf MK, Prabhakar S, Khanduja KL, Anand A (2011). Bacopa monniera Attenuates Scopolamine-Induced Impairment of Spatial Memory in Mice. Evid Based Complement Alternat Med.

[25] Prabhakar S, Saraf MK, Pandhi P, Anand A (2008). Bacopa monniera exerts antiamnesic effect on diazepam-induced anterograde amnesia in mice. Psychopharmacology (Berl).

[26] Saraf M, Prabhakar S, Anand A (2009). Bacopa monniera alleviates N(omega)-nitro-L-arginine arginine-induced but not MK-801-induced amnesia: a mouse Morris watermaze study. Neuroscience.

[27] Minale G, Saesong T, Temkitthawon P, Waranuch N, Nuengchamnong N, Chootip K, Kamkaew N, Kongbangkerd T, Engsuwan J, Ingkaninan K (2021). Characterization of Metabolites in Plasma, Urine and Feces of Healthy Participants after Taking Brahmi Essence for Twelve Weeks Using LC-ESI-QTOF-MS Metabolomic Approach. Molecules.

[28] Mapstone M, Cheema AK, Fiandaca MS, Zhong X, Mhyre TR, MacArthur LH, Hall WJ, Fisher SG, Peterson DR, Haley JM, Nazar MD, Rich SA, Berlau DJ, Peltz CB, Tan MT, Kawas CH, Federoff HJ (2014). Plasma phospholipids identify antecedent memory impairment in older adults. Nat Med.

[29] Sterne JAC, Savović J, Page MJ, Elbers RG, Blencowe NS, Boutron I, Cates CJ, Cheng H, Corbett MS, Eldridge SM, Emberson JR, Hernán Miguel A, Hopewell S, Hróbjartsson Asbjørn, Junqueira DR, Jüni Peter, Kirkham JJ, Lasserson T, Li T, McAleenan A, Reeves BC, Shepperd S, Shrier I, Stewart LA, Tilling K, White IR, Whiting PF, Higgins JPT (2019). RoB 2: a revised tool for assessing risk of bias in randomised trials. BMJ.

[30] Guyatt G, Oxman Andrew D, Akl Elie A, Kunz Regina, Vist Gunn, Brozek Jan, Norris Susan, Falck-Ytter Yngve, Glasziou Paul, DeBeer Hans, Jaeschke Roman, Rind David, Meerpohl Joerg, Dahm Philipp, Schünemann Holger J (2011). GRADE guidelines: 1. Introduction-GRADE evidence profiles and summary of findings tables. J Clin Epidemiol.

[31] Schünemann HJ, Higgins JPT, Vist GE, Glasziou P, Akl EA, Skoetz N, Guyatt GH, On behalf of the Cochrane GRADEing Methods Group (2017). Chapter 14: Completing "Summary of findings" tables and grading the confidence in or quality of the evidence. Cochrane Handbook for Systematic Reviews of Interventions.

[32] Prabhakar S, Vishnu Venugopalan Y, Modi Manish, Mohanty Manju, Sharma Anchal, Medhi Bikas, Mittal B R, Khandelwal Niranjan, Goyal Manoj K, Lal Vivek, Singla Rajesh, Kansal Avinash, Avasthi Ajit (2020). Efficacy of Bacopa Monnieri (Brahmi) and Donepezil in Alzheimer's Disease and Mild Cognitive Impairment: A Randomized Double-Blind Parallel Phase 2b Study. Ann Indian Acad Neurol.

[33] Cicero A, Bove M, Colletti A, Rizzo M, Fogacci F, Giovannini M, Borghi C (2016). Short-term impact of a combined nutraceutical on cognitive function, perceived stress and depression in young elderly with cognitive impairment: a pilot, double-blind, randomized clinical trial. J Prev Alz Dis.

[34] Sadhu A, Upadhyay P, Agrawal A, Ilango K, Karmakar D, Singh GPI, Dubey GP (2014). Management of cognitive determinants in senile dementia of Alzheimer's type: therapeutic potential of a novel polyherbal drug product. Clin Drug Investig.

[35] Raghav S, Singh H, Dalal P, Srivastava J, Asthana O (2006). Randomized controlled trial of standardized Bacopa monniera extract in age-associated memory impairment. Indian J Psychiatry.

[36] Barbhaiya H, Desai RP, Saxena VS, Pravina K, Wasim P, Geetharani P, Allan JJ, Venkateshw K, Amit A (2008). Efficacy and Tolerability of BacoMind®on Memory Improvement in Elderly Participants - A Double Blind Placebo Controlled Study. J of Pharmacology and Toxicology.

[37] Higgins JPT, Deeks JJ (2011). Chapter 7: Selecting studies collecting data. Cochrane Handbook for Systematic Reviews of Interventions.

[38] Goswami S, Saoji A, Kumar N, Thawani V, Tiwari M, Thawani M (2011). Effect of Bacopa monnieri on Cognitive functions in Alzheimer’s disease patients. IJCRIMPH.

[39] Pravina K, Ravindra K R, Goudar K S, Vinod D R, Joshua A J, Wasim P, Venkateshwarlu K, Saxena V S, Amit A (2007). Safety evaluation of BacoMind in healthy volunteers: a phase I study. Phytomedicine.

[40] Parasuraman Subramani, Thing Gan Siaw, Dhanaraj Sokkalingam Arumugam (2014). Polyherbal formulation: Concept of ayurveda. Pharmacogn Rev.

[41] Rosenberg Anna, Solomon Alina, Soininen Hilkka, Visser Pieter Jelle, Blennow Kaj, Hartmann Tobias, Kivipelto Miia, LipiDiDiet clinical study group (2021). Research diagnostic criteria for Alzheimer's disease: findings from the LipiDiDiet randomized controlled trial. Alzheimers Res Ther.

[42] (2018). Clinical investigation of medicines for the treatment Alzheimer’s disease. European Medicines Agency.

[43] Harvey PD, Cosentino S, Curiel R, Goldberg TE, Kaye J, Loewenstein D, Marson D, Salmon D, Wesnes K, Posner H (2017). Performance-based and Observational Assessments in Clinical Trials Across the Alzheimer's Disease Spectrum. Innov Clin Neurosci.

[44] Schneider Lon S, Goldberg Terry E (2020). Composite cognitive and functional measures for early stage Alzheimer's disease trials. Alzheimers Dement (Amst).

[45] Malek-Ahmadi M, Chen K, Perez SE, He A, Mufson EJ (2018). Cognitive composite score association with Alzheimer's disease plaque and tangle pathology. Alzheimers Res Ther.

[46] Ard MC, Edland SD (2011). Power Calculations for Clinical Trials in Alzheimer's Disease. JAD.

[47] Huang Zhiyue, Muniz-Terrera Graciela, Tom Brian D M (2017). Power analysis to detect treatment effects in longitudinal clinical trials for Alzheimer's disease. Alzheimers Dement (N Y).

[48] Alzheimer’s Disease Neuroimaging Initiative.

[49] Committee for Medicinal Products for Human Use (2018). Guideline on the clinical investigation of medicines for the treatment of Alzheimer’s disease. European Medicines Agency.

