# Supplementary material for: Use of Bacopa monnieri in the Treatment of Dementia Due to Alzheimer Disease: Systematic Review of Randomized Controlled Trials
Source: Interact J Med Res. 2022 Aug 1;11(2):e38542. doi: 10.2196/38542 (PMC9379783; doi:10.2196/38542)
Supplement: Multimedia Appendix 1 [file ijmr_v11i2e38542_app1.docx]

**Multimedia Appendix 1.** *Search Strategy.*

No language limit was applied.

| **PubMed Advanced Search Builder** | (((((((((((((Bacopa monnieri)) OR (bacopa monnieri)) OR (hepestris monnieri)) OR (herpestismonniera)) OR (Monieraeuneifolia)) OR (Lysimachia monnieri)) OR (Brahmi)) OR (coastal water hyssop)) OR (water hyssop)) OR (thyme leafed gratiola)) OR (thyme leaved graticule)) AND (((Dementia) OR (cognition)) OR (Alzheimer's disease))) |
| --- | --- |
