# Supplementary material for: Use of Bacopa monnieri in the Treatment of Dementia Due to Alzheimer Disease: Systematic Review of Randomized Controlled Trials
Source: Interact J Med Res. 2022 Aug 1;11(2):e38542. doi: 10.2196/38542 (PMC9379783; doi:10.2196/38542)
Supplement: Multimedia Appendix 2 [file ijmr_v11i2e38542_app2.docx]

**Multimedia Appendix 2.** *Detailed baseline characteristics of the included studies.*

| Table 1 showing demographics , setting of the study and baseline characteristics of the trials included in this systematic review | | | | | | | | | | | | | |
| --- | --- | --- | --- | --- | --- | --- | --- | --- | --- | --- | --- | --- | --- |
| First author  Year  Country | Design | Quality of  Evidence | Objective of the study | Study  population | Intervention in Cases arm | Intervention in Control arm | Number of subjects in intervention arm recruited | Number of subjects in intervention arm that completed trial | Number of controls/comparator arm recruited | Number of controls/comparator arm that completed trial | Baseline characteristic of subjects in intervention arm | Baseline characteristics of subjects in control/placebo arm | Type and timing of Outcome assessment |
| Prabhakar et al., 2020, India | Randomized, double‑blind, parallel‑group, phase‑2 single‑centre clinical trial | Very low | To determine if Bacopa monnieri improved memory of patients with AD and mild cognitive impairment-AD compared to Donepezil. | Patients aged >50 years, diagnosed with  MCI‑AD or AD (Dubois criteria) with modified Hachinski  ischemic scale score of less than 5 points, and mini‑mental  status examination (MMSE) more than 10 | *Bacopa monnieri* - once daily at a dose of 300 mg for 12 months. | Donepezil - at a dose of 10 mg once daily for 12 months | 17 | 13 | 17 | 8 | Gender – male – 13 (76.5%)  Age - 70.18 ± 6.73  AD – 05(29.4%0  MCI-AD – 7 (41.2%)  HTN – 10 (58.8%)  DM – 7 (41.2%)  Median Duration of disease – 12 (IQR: 12-30)  ADAS - 12.61±1.03  CDR – 0.67±0.07  Animal Naming test – 7.35±0.77  COWAT – 5.55±0.62  Quality of Life (Patient) – 33.88±1.02  Quality of Life (Informant) – 31.88±1.02  ADL – 56.88±2.83  MMSE - 23.76±0.97  PGI Memory Scale – 54.94±3.60  Remote memory – 4.82±0.37  Recent Memory – 3.82±0.38  Mental Balance – 6.23±0.63  Attention and concentration – 8.05±0.59  Delayed Recall – 5.23±0.53  Immediate recall – 5.88±0.55  Retention for similar pairs – 4.05±0.29  Retention for dissimilar pairs – 6.05±0.90  Visual Retention – 4.23±0.87  Visual Recognition – 6.53±0.58 | Gender - Male 09 (52.9%)  Age – 68.71± 9.54  AD – 10 (58.8%)  MCI AD - 12 (70.6%)  HTN – 5 (29.4%)  DM – 4 (23.5%)  Median Duration of disease – 24 (IQR: 12-42)  ADAS - 17.09±1.63  CDR – 0.85±0.09  Animal Naming test – 6.35±0.65  COWAT – 3.23±0.59  Quality of Life (Patient) – 35.18±1.96  Quality of Life (Informant) – 34.06±1.90  ADL – 53.76±2.85  MMSE – 18.06±1.50  PGI Memory Scale – 41.11±4.28  Remote memory – 4.00±0.36  Recent Memory – 2.82±0.39  Mental Balance – 3.76±0.77  Attention and concentration – 7.06±0.51  Delayed Recall – 4.11±0.57  Immediate recall – 4.24±0.60  Retention for similar pairs – 3.29±0.28  Retention for dissimilar pairs – 4.18±0.87  Visual Retention – 2.65±0.75  Visual Recognition – 5.00±0.70 | Alzheimer disease assessment scale – Cognitive subscale (ADAS-Cog), PGI memory scale, verbal fluency-controlled oral word test, animal names test, quality of life-AD, activities of daily living inventory, compliance to treatment and adverse events assessed at baseline, 3, 6, 9 and 12 months of treatment in both the groups. |
| Cicero et al., 2017, Italy | Double bind, cross-over placebo controlled randomized trial. | Very low | To evaluate a combination of agents over 2 months on improvement of cognitive functions in elderly patients | 30 elderly subjects with basal Mini-Mental State Examination score between 20 and 27 and self-perceived cognitive  decline. | Intervention arm received a combined nutraceutical containing Bacopa monnieri dry extract 320 mg, L-Teanina  100 mg, Crocus sativus 30 mg, some vitamins (Vitamin B6 9,5 mg, Biotine 450 mcg, Folic acid 400 mcg, Vitamin B12 33 mcg,  Vitamin D 25 mcg) and Cupper 2 mg.  1 capsule once daily for 8 weeks | An indistinguishable placebo-  1 capsule once daily for 8 weeks | 15 | 15 | 15 | 15 | Age – 66 ±3  Self-perceived cognitive decline without known diagnosis of cognitive decline nor dementia.  All received  ACE-inhibitors/sartans and statins.  MMSE – 23.1±0.9  PSQ Index - 2.7±0.4  SRDS- 42.8±8.4 | Age - 65±4  Self-perceived cognitive decline without known diagnosis of cognitive decline nor dementia.  All received  ACE inhibitors /sartans and statins.  MMSE - 23.2±1.1  PSQ Index - 2.6±0.8  SRDS - 43.6±9.3 | Patients were evaluated with Mini-Mental State Examination (MMSE), Perceived Stress Questionnaire (PSQ) and  Index and Self-Rating Depression Scale (SRDS) at baseline and at 2 months post treatment |
| Sadhu et al., 2014 | Randomized double-blind placebo- and active controlled trial | Very low | To evaluate the efficacy of a polyherbal  (test) formulation on cognitive functions, inflammatory  markers and oxidative stress in healthy elderly as well as  senile dementia of Alzheimer’s type (SDAT) patients. | Patients of Senile dementia of Alzheimer’s type (SDAT) with an age range of 60–75 years. | *Group C:* SDAT patients – given  standard drug donepezil 10 mg, twice daily  *Group D:* SDAT patients – given  test formulation 500 mg twice daily.  All for 12 months  Test formulation contained -  extracts of Bacopa monnieri (whole plant), Hippophae  rhamnoides (leaves and fruits) and Dioscorea  bulbifera (bulbils) | *Group A:* healthy elderly subjects – given  Placebo  *Group B:* healthy elderly subjects -  given test formulation | 123  Group C+D | 104  Group  C+D  Group C – 43  Group D - 61 | 109  Group A+B | 97  Group A+B  Group A-41  Group B-56 | Gender – not mentioned  Age – Range 60-75 years  Mean age not provided  Symptoms -Aggression – 24 %  Sleep disturbance 20%  Communication difficulty 0%  Depression 25%  Psychotic features 9%  Homocysteine (nmol/L) - 38.42 ±4.16  CRP (mg/L)- 6.23 ±1.09  IL-6 (pg/ml) - 4.12 ±1.06  TNF-a (pg/ml) - 1124.06   ±105.87    *(For Group C+D)* | Gender – Not mentioned  Age – Range 60-75 years  Mean age not provided  Symptoms -Aggression 62%  Sleep disturbance 71%  Communication difficulty 79%  Depression 68%  Psychotic feature 67%  Homocysteine (nmol/L)-21.99±2.85  CRP (mg/L)- 2.38±0.76  IL-6 (pg/ml) - 2.23±0.41  TNF-a (pg/ml) -483.80±85.64  *(For Group A+B)* | Cognitive functions  assessed at baseline and every 3 months by MMSE, DSS; WAIS, Adult Intelligence Scale—  Revised, digital  memory apparatus—Medicaid systems, Chandigarh,  India, FAQ and depression (geriatric depression scale)  scores.  Inflammatory markers and level of oxidative  stress were analysed using standard biochemical tests at baseline and every 3 months. |
| Raghav et al., 2006,  India | Double-blind, placebo-controlled randomized study | Very low | To study the efficacy of standardized Bacopa monniera extract (SBME) in subjects with age-associated  memory impairment (AAMI) without any evidence of dementia or psychiatric disorder. | Included participants were adults above 55 years of age with memory loss in daily activities having a logical subset score less than 6 on the Wechsler Memory scale. Patients with MMSE score more than 24 were excluded. | 125 mg of SBME twice a day for a period of 12 weeks followed by a placebo for another  4 weeks (total duration of the trial 16 weeks). | Placebo twice a day for a period of 12 weeks followed again by a placebo period of another  4 weeks (total duration of the trial 16 weeks). | 20 | 18 | 20 | 17 | Gender –  Male - 19 (95%);  Age – 55-60 years -predominant age group  Mean age not provided  Duration of memory loss –  Cases - <2 years – 12(60%), >2 years – 8(40%) | Gender –  Male - 18 (65%)  Age – 55-60 years -predominant age group  Mean age not provided  Duration of memory loss –  Controls - < 2 years- 11(55%) , >2 years – 9(45%) | Each subject was evaluated for cognition on a battery of tests  comprising mental control, logical memory, digit forward, digit backward, visual reproduction and paired associate learning at baseline, 4, 8, 12 and 16 weeks. |
| Barbhaiya et al, 2008 | Double-blind, placebo-controlled randomized study | Very low | To evaluate the efficacy and tolerability of BacoMind on memory improvement in elderly patients. | Elderly individuals with age between 50 and 75 years with memory impairment for 1 year | One capsule daily of BacoMind containing 450 mg of standardised extract of Bacaopa monnieri was given for 12 weeks. | Placebo which was similar to active drug was given daily for 12 weeks | NA | 23 | NA | 21 | Gender Male: 14/23  Age (mean±SD): 65.52±8.79)  (only per-protocol data available)  Attention  Digit span forward: 5.26±0.18  Digit span backward  3.48±0.21  Digit cancellation test time (sec)  179.48±8.94  Digit cancellation test Error  2.48±0.63  Serial subtraction time (sec)  79.57±7.81  Serial subtraction Error  1.48±0.22  Memory Verbal  List learning immediate recall (IR)  4.39±0.34  List learning delayed recall(DR)  1.78±0.37  Paired associates similar (IR)  4.65±0.10  Paired associates dissimilar (IR)  8.52±0.59  Paired associates similar (DR)  4.09±0.15  Paired associates dissimilar (DR)  2.39±0.29  Passages Immediate Recall (IR)  13.61±0.79  Passages Delayed Recall (DR)  12.04±0.90  Visual Retention I  7.70±0.77  Visual Retention II  8.78±0.27  Speed of Information Processing  Digit symbol  24.39±2.75 | Gender: Male:15/21  Age (mean±SD): 62.86±10.61)  (only per-protocol data available)  Attention  Digit span forward: 4.86±0.28  Digit span backward  3.33±0.23  Digit cancellation test time (sec)  167.00±11.48  Digit cancellation test Error  3.29±0.97  Serial subtraction time (sec)  52.14±6.83  Serial subtraction Error  0.90±0.28  Memory Verbal  List learning immediate recall (IR)  4.48±0.29  List learning delayed recall (DR)  2.24±0.34  Paired associates similar (IR)  4.52±0.15  Paired associates dissimilar (IR)  8.67±0.56  Paired associates similar (DR)  4.14±0.21  Paired associates dissimilar (DR)  2.52±0.30  Passages Immediate Recall (IR)  14.43±0.94  Passages Delayed Recall (DR)  14.00±1.01  Visual Retention I  8.43±0.76  Visual Retention II  8.71±0.27  Speed of Information Processing  Digit symbol  32.29±3.12 | Each subject was evaluated using a battery of neuropsychological tests in three main domains (attentions, memory verbal and speed of information processing) at baseline, 12 and 24 weeks |
| AD- Alzheimer’s Disease, ACE- Angiotensin Converting Enzyme, ADAS-Cog - Alzheimer disease assessment scale – Cognitive subscale, ADL – Activities of Daily Living, CDR – Clinical Dementia Rating, COWAT – Controlled Oral Word Association test, CRP – C Reactive Protein, DM – Diabetes Mellitus, DSS - digital symbol substitution, FAQ - functional activity questionnaire, HTN – Hypertension, MMSE - mini‑mental status examination, MCI – Mild Cognitive Impairment, PGIMS -PGI Memory Scale, PGI - Post Graduate Institute, PSQ - Perceived Stress Questionnaire, SDAT - Senile dementia of Alzheimer’s type, SRDS- Self-Rating Depression Scale, WAIS – Wechsler Adult Intelligence Scale | | | | | | | | | | | | | |
