# Supplementary material for: Use of Bacopa monnieri in the Treatment of Dementia Due to Alzheimer Disease: Systematic Review of Randomized Controlled Trials
Source: Interact J Med Res. 2022 Aug 1;11(2):e38542. doi: 10.2196/38542 (PMC9379783; doi:10.2196/38542)
Supplement: Multimedia Appendix 3 [file ijmr_v11i2e38542_app3.docx]

| **Multimedia Appendix 3. Quality of evidence, outcomes, and adverse events.** | | | | | | | | |
| --- | --- | --- | --- | --- | --- | --- | --- | --- |
| **Table 2 showing the important outcome measures and adverse events reported in the studies included in this systematic review** | | | | | | | | |
| **First author**  **Year**  **Country** | **Participant group** | **Summary of relevant outcome measures** | **Subset of relevant outcome measures** | **Between group parameters relevant to the pertinent outcome** | **Mean difference, 95% CI** | **Lost to follow-up/ dropouts and handling of missing data** | **Adverse events reported** | **Mortality if any** |
| Prabhakar et al., 2019, India | Comparison between  subjects in donepezil and Brahmi arm | A significant difference was noted in the change in PGI memory scale score between the two arms (Donepezil and Brahmi) at 12 months. Donepezil arm had lesser progression | PGI Memory Scale score | 12 months  Donepezil – - 0.46 (10.96)  vs  Brahmi -7.94 (10.96); mean difference -8.40 | 0.04 | Donepezil arm - Four (24%)  Bacopa arm - Nine (53%).  At the end of 12 months of follow up.  Missing data was handled by multiple imputations due to loss to follow up. | No significant differences in the number of patients in two arms who experienced side effects. No major adverse events reported. | There were three deaths (two in donepezil and one in Brahmi arm) reported due to myocardial infarction. |
|  |  | No difference in rate of change in ADAS-Cog score between the Brahmi arm and donepezil arm, at any of the prespecified time points (3, 6, 9 and 12 months) from baseline. | ADAS-Cog score | 12 months  Donepezil - 2.27(5.65)  vs  Brahmi - 0.51(5.65)  mean difference – 1.76 | 0.39 |  |  |  |
|  |  | No significant difference in the analysis of the secondary outcomes CDR, MMSE, ADCS‑ADL, QOL, animal naming test, and controlled oral word association test) | CDR | 12 months  Donepezil – - 2.27 (0.47)  vs  Brahmi - -0.07 (0.47); mean difference -0.20 | 0.23 |  |  |  |
|  |  |  | MMSE | 12 months  Donepezil – 1.59 (4.45)  vs  Brahmi -2.31 (4.45); mean difference - -0.72 | 0.66 |  |  |  |
|  |  |  | ADCS‑ADL | 12 months Donepezil – 2.35 (11.7)  vs  Brahmi -4.47 (11.7); mean difference - -2.12 | 0.60 |  |  |  |
|  |  |  | QOL | 12 months  Donepezil – 0.29 (4.99)  vs  Brahmi – 0.37 (4.99); mean difference - -0.08 | 0.96 |  |  |  |
|  |  |  | Animal naming test | 12 months  Donepezil – 0.72 (2.52)  vs  Brahmi -0.62 (2.52); mean difference – 0.09 | 0.91 |  |  |  |
|  |  |  | Controlled oral word association test (COWAT) | 12 months  Donepezil – 0.26 (2.10)  vs  Brahmi -0.55 (2.10); mean difference - -0.29 | 0.70 |  |  |  |
|  |  | MMSE at 3 months showed significant improvement in donepezil arm. | MMSE | 3 months  Donepezil – 0.72 (3.13)  vs  Brahmi - -2.02 (3.13); mean difference – 2.74 | 0.02 |  |  |  |
| Cicero et al., 2017, Italy | Intervention arm vs placebo | Significant improvements in MMSE, PSQ index in the Brahmi formulation arm compared to placebo. | MMSE | 2 months  Brahmi – 24.5±1.0  vs  placebo – 23.1±0.9 | <0.05 | No lost to follow-up/dropouts reported | Only one patient claimed aftertaste after active product intake.  No other adverse event reported | Nil |
|  |  |  | PSQ Index | 2 months  Brahmi – 2.2±0.7  vs  placebo – 2.4±0.9 | <0.05 |  |  |  |
|  | Intervention arm and placebo versus their respective baseline | Significant improvements in SRDS score in both Brahmi formulation arm and placebo at 2 months compared to baseline. | SRDS score | Brahmi group-  Baseline – 42.8±8.4  vs  at 2 months – 37.1±7.6  Placebo group –  Baseline – 43.6±9.3  vs  at 2 months – 40.9±8.8 | <0.05 |  |  |  |
| Sadhu et al., 2014, India | In patients with SDAT | Test formulation at 12 months was effective in improving cognitive functions in the SDAT patients, when compared to the donepezil-treated group, as determined by the following – DSS scores, word recall immediate, and attention span scores | DSS scores | Group D at 12 months - 38.984±3.016  vs  Group C at 12 months - 35.852±4.906 | 0.0001 | Group A – 10 (19.6%)  Group B – 2(3.4%)  Group C – 15 (24.8%)  Group D – 4 (6.15%)  All dropouts are lost to follow up.  No information on how missing data was accounted for in the final analysis. | Dropout rates lower for the test formulation group compared to corresponding placebo or donepezil 🡪 indicating the test formulation is safe and well tolerated  Adverse events reported in trial –  nausea, constipation and/or drowsiness were transient and  mild in severity.  Incidence or assessment scales not provided | Nil |
|  |  |  | word recall immediate scores | Group D at 12 months - 3.594±1.003  vs  Group C at 12 months - 2.794±0.593 | <0.0001 |  |  |  |
|  |  |  | attention span scores | Group D at 12 months – 4.918±1.239  vs  Group C at 12 months - 4.396±0.913 | 0.0208 |  |  |  |
|  |  | Significant improvement in FAQ and depression also | FAQ score | Group D at 12 months - 11.873±2.751  vs  Group C at 12 months - 9.801±1.458 | < 0.0001 |  |  |  |
|  |  |  | Depression | Group D at 12 months - 16.387±2.116  vs  Group C at 12 months -21.006±2.778 | < 0.0001 |  |  |  |
|  |  | No significant differences were observed in the MMSE, and word recall delayed scores. | MMSE | Group D at 12 months - 7.914±2.106  vs  Group C at 12 months – 7.882±1.956 | 0.9375 |  |  |  |
|  |  |  | Word recall delayed | Group D at 12 months - 2.049±0.229  vs  Group C at 12 months – 1.884±0.597 | 0.0520 |  |  |  |
|  |  | Marked reduction in  inflammation and oxidative stress in the SDAT patients treated with the test formulation compared to the donepezil-treated group | Homocysteine in nmol/L | Group D at 12 months- 30.22±3.87  vs  Group C at 12 months – 44.73±7.11 | <0.0001 |  |  |  |
|  |  |  | CRP in mg/L | Group D at 12 months- 4.751±1.149  vs  Group C at 12 months – 5.887±1.049 | <0.0001 |  |  |  |
|  |  |  | TNF -alpha in pg/ml | Group D at 12 months-1139.45±198.87  vs  Group C at 12 months- 1598.77±298.52 | <0.0001 |  |  |  |
|  |  |  | Superoxide dismutase in U/g Hb | Group D at 12 months1145.92±228.75  vs  Group C at 12 months – 1296.32 ±225.72 | 0.0013 |  |  |  |
|  |  |  | Glutathione peroxidase  U/g Hb | Group D at 12 months- 20.78±3.14  vs  Group C at 12 months – 25.99±4.11 | <0.0001 |  |  |  |
|  |  |  | Glutathione  U/g Hb  (increase) | Group D at 12 months- 9.358±2.139  vs  Group C at 12 months – 6.831±1.139 | <0.0001 |  |  |  |
|  |  |  | Thiobarbituric acid derivatives reactive subsatances  TBARS | Group D at 12 months- 131.62±29.68  vs  Group C at 12 months – 176.40±68.11 | <0.0001 |  |  |  |
|  | In healthy elderly subjects treated with the test formulation | Healthy elderly subjects treated with the test formulation showed a significant improvement in  in cognitive measures  (MMSE, DSS, word recall delayed) | MMSE | Group B at 12 months- 18.14±4.02  vs  Group A at 12 months – 14.90±4.32 | 0.0003 |  |  |  |
|  |  |  | DSS | Group B at 12 months- 49.01±12.13  vs  Group A at 12 months – 41.32±8.63 | 0.0008 |  |  |  |
|  |  |  | Word recall delayed | Group B at 12 months- 4.002±0.754  vs  Group A at 12 months – 3.128±0.759 | <0.0001 |  |  |  |
|  |  | Significant improvement in word recall immediate, attention span, FAQ and depression scores at 12 month.  However in the published manuscript, these outcomes have been categorized as being ‘non-significant’. | Word recall immediate | Group B at 12 months- 4.012±1.004  vs  Group A at 12 months – 3.718±0.96 | 0.1500 |  |  |  |
|  |  |  | Attention span | Group B at 12 months- 9.382±1.942  vs  Group A at 12 months – 6.874±1.119 | <0.0001 |  |  |  |
|  |  |  | FAQ | Group B at 12 months- 17.316±3.014  vs  Group A at 12 months – 15.913±2.557 | <0.0001 |  |  |  |
|  |  |  | Depression score | Group B at 12 months- 9.184±3.114  vs  Group A at 12 months – 13.087±2.856 | <0.0001 |  |  |  |
|  |  | Reduction in levels of inflammatory markers | Homocysteine in nmol/L | Group B at 12 months- 12.823±2.104  vs  Group A at 12 months – 24.813±3.106 |  |  |  |  |
|  |  |  | CRP in mg/L | Group B at 12 months- 3.0931±1.004  vs  Group A at 12 months – 4.612±1.106 |  |  |  |  |
|  |  |  | IL -6 in pg/ml | Group B at 12 months- 1.332±0.801  vs  Group A at 12 months – 2.941±0.583 |  |  |  |  |
|  |  |  | TNF -alpha in pg/ml | Group B at 12 months -584.730±114.902  vs  Group A at 12 months – 811.693±131.685 |  |  |  |  |
|  |  |  | Superoxide dismutase in U/g Hb | Group B at 12 months- 738.73±125.01  vs  Group A at 12 months-1189.36±189.57 |  |  |  |  |
|  |  | Improvement in oxidative stress | Glutathione peroxidase  U/g Hb | Group B at 12 months- 16.142±3.014  vs  Group A at 12 months – 19.775±3.914 | <0.0001 |  |  |  |
|  |  |  | Glutathione  U/g Hb  (increase) | Group B at 12 months- 9.868±1.451  vs  Group A at 12 months – 7.139±1.908 | <0.0001 |  |  |  |
|  |  |  | Thiobarbituric acid derivatives reactive subsatances  TBARS | Group B at 12 months- 106.64±34.90  vs  Group A at 12 months – 139.94±34.82 | <0.0001 |  |  |  |
| Raghav et al., 2006, India | SBME group vs control group | SBME produced significant improvement on mental control, logical memory and paired associated  Learning, digit forward subset and total score during the 12-week drug therapy. | Logical memory subtest | 12 -weeks  SBME group – 8.7 ± 2.1  Vs  placebo – 6.7 ± 1.6 | P < 0.01 | No information on the lost-to follow-up subjects and how the missing data was accounted for in the final analysis. | Adverse events –  SBME group – maculo-papular rash in one patient – aetiology not known  Placebo group – one patient had diarrhoea, two had headache, all self-resolved | Nil |
|  |  |  | Mental Control subset | 12 -weeks  SBME group – 8.6 ± 0.6  Vs  placebo - 8.0 ± 0.4 | P < 0.01 |  |  |  |
|  |  |  | Digit Forward Subset | 12 -weeks  SBME group – 7.0 ± 0.7  Vs  placebo – 6.8 ± 0.7 | P < 0.01 |  |  |  |
|  |  |  | Paired Associate Learning subset | 12 -weeks  SBME group – 18.1 ± 2.3  Vs  placebo – 14.5 ± 1.8 | P < 0.05 |  |  |  |
|  |  |  | Total Score | 12 -weeks  SBME group – 70.2 ± 4.8  Vs  placebo – 64.6 ± 4.8 | P < 0.01 |  |  |  |
| Barbhaiya et al, 2008, India | Bacopa monnieri group vs placebo group | Significant interaction effects between group and time were observed in digit span backward test, list learning delayed recall test, paired associates dissimilar delayed recall test, and in visual retention-I test. | Digit span backward (group x time) | 24 - weeks  Brahmi group – 3.26±0.21  Vs  Placebo group – 3.48±0.25 | 0.22 [0.08, 0.36] | 32% loss to follow up (21 out of 65 patients) out of which 15 patients were removed as ‘outliers’.  The authors neither gave any definition for “outliers” nor any explanation for their removal.  In another part of the results sections, a different scenario of drop-outs was given as below:  One participant dropped out from the placebo group at two weeks .  One from each of the Brahmi and placebo group were withdrawn from the study due to medical reasons unrelated to therapy.  In either scenario, a per protocol analysis was used and it was not mentioned how missing data was handled. | No serious adverse events reported.  Only described as ‘no mild moderate or mild adverse events reported by participants or explored by investigator’, but the exact nature of the adverse events and number affected not mentioned | Nil |
|  |  |  | List learning delayed recall (group x time) | 24 weeks  Brahmi group – 3.17±0.31  Vs  Placebo group – 2.19±0.28 | 0.98 [0.8, 1.2] |  |  |  |
|  |  |  | Paired associates  Dissimilar delayed recall test (group x time) | 24 weeks  Brahmi group – 3.26±0.31  Vs  Placebo group – 2.38±0.33 | 0.88 [0.68, 1.07] |  |  |  |
|  |  |  | Visual Retention I | 24 weeks  Brahmi group – 8.57±0.82  Vs  Placebo group – 8.67±0.33 | 0.1 [-0.28,0.48] |  |  |  |
| AD- Alzheimer’s Disease, ACE- Angiotensin Converting Enzyme, ADAS-Cog - Alzheimer disease assessment scale – Cognitive subscale, ADL – Activities of Daily Living, CDR – Clinical Dementia Rating, COWAT – Controlled Oral Word Association test, CRP – C Reactive Protein, DM – Diabetes Mellitus, DSS - digital symbol substitution, FAQ - functional activity questionnaire, HTN – Hypertension, IL6 -Interleukin 6, MMSE - mini‑mental status examination, MCI – Mild Cognitive Impairment, PGIMS -PGI Memory Scale, PGI - Post Graduate Institute, PSQ - Perceived Stress Questionnaire, QOL – Quality of Life, SBME - standardized Bacopa monniera extract, SDAT - Senile dementia of Alzheimer’s type, SRDS- Self-Rating Depression Scale, TNF – Tumour Necrosis Factor, WAIS – Wechsler Adult Intelligence Scale | | | | | | | | |
