# Supplementary figures and images for: Use of Bacopa monnieri in the Treatment of Dementia Due to Alzheimer Disease: Systematic Review of Randomized Controlled Trials
Source: Interact J Med Res. 2022 Aug 1;11(2):e38542. doi: 10.2196/38542 (PMC9379783; doi:10.2196/38542)

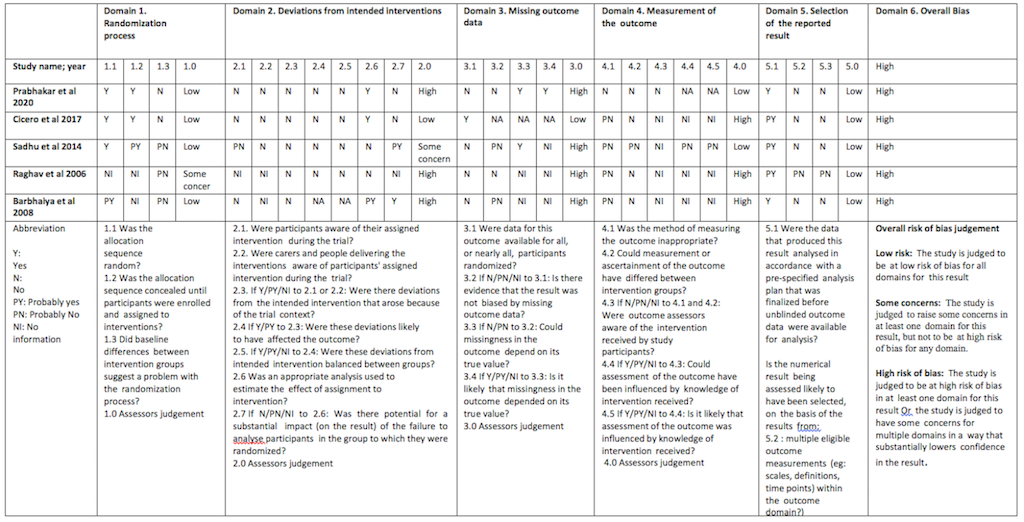

Supplement: Multimedia Appendix 4 [file ijmr_v11i2e38542_app4.png]
